# Queer intimacy under constraint in Ruth Vanita’s *A Slight Angle*

**DOI:** 10.3389/fsoc.2026.1805544

**Published:** 2026-08-25

**Authors:** N. Abarna, B. Sivakami

**Affiliations:** School of Social Sciences and Languages, Vellore Institute of Technology, Chennai, India

**Keywords:** colonial legacies, queer (LGBTQ), queer intimacy, queer spatiality, queer temporality, Indian Queer Literature

## Abstract

This paper investigates queer intimacy under constraint in Ruth Vanita’s *A Slight Angle*, a novel that reimagines same-sex desire within colonial India’s disciplinary structures of pedagogy, domesticity, and social regulation. Drawing on Foucauldian theories of disciplinary power, Ahmed’s queer phenomenology of spatial orientation, and Halberstam’s framework of queer temporality, it examines how constraint paradoxically generates attachment and belonging. Through interpretive qualitative methodology, specifically close textual analysis of minor gestures, gazes, silences, sensory details, and liminal encounters, the study reveals that classrooms, letters, seaside homes, and thresholds function as sites where regulation and desire mutually constitute one another. Key findings indicate that queer life endures not despite but through colonial mechanisms, transforming control into affective memory and resilient kinship networks. These intimacies persist as emotional legacies, informing contemporary queer belonging amid India’s uneven legal and social landscapes.

## 1 Introduction

Queer life in India has long been shaped by the intersecting forces of colonialism, law, social hierarchy, and culture. Section 377 of the Indian Penal Code (a colonial statute) extended the state’s authority into the most intimate realms of life, criminalising consensual same-sex activities for over a century. The Supreme Court of India advanced gender self-determination by recognising a third gender in National Legal Services Authority v. Union of India (2014) and upheld sexual autonomy by decriminalising consensual same-sex relations in Navtej Singh Johar v. Union of India (2018). Yet these legal milestones remain incomplete. Their limitations became evident in Supriyo @ Supriya Chakraborty & Anr. v. Union of India (2023), where the Court denied same-sex couples the right to marry, granting partial legal protections while withholding the social legitimacy and material security of marital recognition. Even in a progressive state like Tamil Nadu, which implements transgender policies guaranteeing employment, education, and anti-discrimination measures (Government of Tamil Nadu, 2025), same-sex couples remain excluded from marital frameworks. This unevenness highlights how queer rights in India are distributed differentially across gender identities and sexualities, rather than being extended uniformly. Law thus emerges as a contested terrain where freedom, visibility, vulnerability, and belonging are continually negotiated. As Bhan (2005) argues, queer claims to rights are articulated within a legal system whose language of legitimacy is deeply entangled with caste, class, and respectability. Legal recognition, therefore, often requires conformity to normative ideals even as it promises emancipation. Queer political engagement in India is consequently marked by tension, as it demands access to rights while remaining constrained by the hierarchies that structure recognition itself.

This paper argues that everyday negotiations of space across both private and public domains are central to understanding how queer belonging is constituted in colonial India. Reading Ruth Vanita’s *A Slight Angle* as a literary archive situated within South Asian queer scholarship, the study draws on regionally grounded work alongside broader queer theoretical frameworks to examine how intimacy is produced, regulated, and reimagined across pedagogical, domestic, and liminal spaces.

Ruth Vanita is a scholar of South Asian literature and queer history whose work has played a foundational role in documenting the historical presence of same-sex desire in India. She is widely known for *Same-Sex Love in India: Readings from Literature and History* (2000), co-edited with Saleem Kidwai, which assembles literary and historical references to same-sex relationships across more than two millennia of South Asian cultural history. Alongside her scholarly work, Vanita has written fiction that imaginatively reconstructs queer lives within historical contexts. Her novel *A Slight Angle* (2024) draws on this longstanding engagement with textual and cultural archives to explore how intimacy, discipline, and belonging intersect within the social environments of colonial India.

A Slight Angle is set in early twentieth-century colonial India and follows several intersecting relationships among a small circle of characters. One strand of the narrative centres on Sharad, a school student who develops a deep admiration for his English teacher Abhik, a young instructor only a few years older than his pupils. Their connection grows through classroom interactions, theatre rehearsals, and shared discussions of literature. Alongside this storyline is the relationship between Sharad’s sister Kantha and Robin Singh, a Christian man whom she hopes to marry despite the opposition of her caste-conscious Hindu family. Sharad quietly assists his sister in sustaining this relationship, which brings him into contact with Robin’s sister Sheela. Sheela had previously lived in an ashram devoted to a life of service but later forms a close relationship with Rita, a cosmopolitan Jewish actress known for her lively recitations of film dialogues and theatrical expressions. As the narrative moves across schools, homes, train journeys, and seaside residences in Bombay, these characters’ lives gradually intersect. Through these intertwined stories, the novel depicts both male and female same-sex relationships, including a gay relationship between Sharad and Abhik and a lesbian relationship between Sheela and Rita. Through Rita’s public connections and social gatherings, Sharad and Abhik encounter one another again after a long period of separation, leading to a moment in which Abhik finally reveals letters he had written but never sent to Sharad during the years they were apart.

Classrooms and homes emerge as sites where disciplinary power coexists with emotional attachment. At the same time, colonial regimes of language, literature, and aesthetics function simultaneously as instruments of control and as conditions for desire. These entanglements suggest that regulation and intimacy are not oppositional forces but mutually constitutive processes. Queer intimacy, rather than arising outside structures of power, is formed through the very mechanisms that seek to constrain it. The intimacies shaped under colonial discipline persist as affective inheritances, continuing to inform how queer belonging is imagined, negotiated, and lived in contemporary India.

Vanita’s longstanding engagement with South Asian queer history also shapes *A Slight Angle* as more than a work of historical fiction. Rather than reconstructing the colonial past through documentary evidence alone, the novel draws on historically grounded social, cultural, and literary contexts to imagine how queer intimacy might have been experienced within everyday institutional life. In this sense, the novel functions as a literary archive that renders affective, spatial, and interpersonal dimensions of colonial queer existence analytically visible while remaining attentive to the imaginative possibilities of fiction. This study approaches these literary spaces as representations of historically organised social relations rather than as fictional settings alone. The classrooms, homes, theatrical spaces, and transitional environments depicted in *A Slight Angle* are analysed as cultural sites through which colonial institutions organised intimacy, authority, and belonging. Approaching these spaces sociologically allows the novel to illuminate broader processes through which institutional regulation shaped everyday queer life beyond the experiences of individual characters.

## 2 Beyond the law: queer intersections and spaces in India/literature review

Beyond the struggle for legal rights, queer life in India is mediated through the interlocking hierarchies of caste, class, religion, and geography. Although the queer movement has gained increased public visibility in recent years, it continues to centre the experiences of elite, urban, English-speaking, dominant-caste individuals, leaving those from marginalised backgrounds structurally overlooked and unheard (Bacchetta, 2002; Ponniah and Tamalapakula, 2020). Understanding the circulation of Western identity frameworks in global contexts requires recognising the methodological and thematic convergence between postcolonial and queer studies (Hawley, 2001). Global queer paradigms are refracted through local social formations, producing positionalities that are contextually embedded and relational rather than universal (Gopinath, 2005).

Aesthetic norms within urban queer spaces can reproduce caste and class hierarchies, marginalising Dalit and lower-caste queer individuals (Pradeep, 2025). The intersection of religion and caste shapes conditions of queer legibility, which in turn affect the ability to inhabit public sites of queer sociality, thereby delimiting which relationships can be recognised and which must remain concealed (Ponniah and Tamalapakula, 2020). The relationship between queer subjectivities in India and power is highly complicated; homonationalism plays a significant role in shaping how recognition is granted, what it impacts, and who experiences a sense of belonging to what extent (Puar, 2007). Shahani’s (2020) *Queeristan* discusses in detail how abuse within biological families forces many queer Indians to seek support within chosen kinship networks.

Queer affiliation within activist and community settings is rooted in a process of ethical self-fashioning. Here, ethics operates as a relational practice, involving an iterative reinterpretation of intimacy, obligation, and kinship to accommodate queer existence amid ongoing negotiations with family, community, and moral expectation (Dave, 2012). Taken together, these dynamics suggest that freedom in the Indian queer context does not emerge from liberal notions of individual autonomy but from moral reasoning rooted in the lived ethics of everyday relationships. Pradeep (2025) argues that the regulation of sexuality in India is organised around caste, patriarchal, and nationalist moralities that establish hierarchies of desire, restricting intimacy, endogamy, and sexual freedom. These regulatory structures operate both within heteronormative familial formations and within queer community spaces themselves. Desire, in this framework, is neither a purely individual nor a private impulse but a social relation negotiated within systems of purity, legitimacy, and belonging. Queer spatiality, therefore, cannot be confined to urban or activist sites alone as it emerges wherever normative regimes of discipline and morality are questioned or strategically reworked. Queer women and non-binary individuals, in particular, cultivate networks of safety, visibility, and care by mobilising both public and virtual platforms, including online collectives and social media spaces (Sahgal, 2021).

The historical record of Indian queerness directly challenges the myth of homosexuality as a western import, which acts as a critical base for decolonising contemporary queer identity and spatial politics. In their 2000 work, *Same-Sex Love in India*, Ruth Vanita and Saleem Kidwai constructed a compelling counter-narrative to colonial discourse. Their extensive archive, drawing on over two millennia of literary evidence, proves that diverse forms of same-sex love were acknowledged in pre-colonial India. The suppression of queer lives and histories creates a contemporary archive of feelings that is continuously shaped by historical loss and trauma. This recognises historical loss and trauma not as past events but as active forces influencing present-day queer affect (Cvetkovich, 2003). Literature becomes vital here as an alternative archive, as seen in R. Raj Rao’s *The Boyfriend*, which vividly portrays gay subculture in 1990s Bombay. It showcases how specific urban locales, such as Churchgate Station and gay discos, become ‘queer spaces’ that offer comfort while highlighting the precarity of life in a heteronormative society (Rao, 2003). The queer literary explorations that interact with a cultural context are still informed by colonial history. These explorations work to challenge the historical marginalisation of non-western sexualities and contest ongoing normative pressures (Gopinath, 2005). In a global capitalist system that demands a linear progression, non-market-driven queer lives are often positioned out of time (Rao, 2020). This context helps explain why certain narratives emphasise yearning and potentiality over immediate gratification. The analysis draws insight from Nizara Hazarika's (2022) work on transgressive spatialities and liminality, which the author observes in contemporary queer narratives from non-metropolitan Indian contexts.

The way social categories shape city space helps explain how queer mobility works in fear and the strategy involved. The spatial negotiation of sexuality has also been theorised through the metaphor of the “closet,” understood not only as a metaphor for secrecy but as a spatial structure that organises visibility and concealment in everyday environments. Brown (2005) further demonstrates how the closet operates as a spatial arrangement embedded within everyday social environments, structuring when and where queer identities become visible. Queer people mentally map urban environments as constellations in response to urban precarity, using these fragmented experiences not only to navigate but also to build resilience beyond mere physical safety (Gieseking, 2020). Hanhardt (2013) mentions that the pursuit of safe spaces in urban areas, particularly when driven by policing and privatisation efforts, can paradoxically reinforce existing racial and class inequalities.

The home emerges as a transformative space that stands in contrast to the restrictions of public life. For queer couples, it offers a fragile sense of safety where intimacy and belonging are negotiated through small, everyday gestures of care, despite the intruding gaze of family and society. By simply doing the dishes or sharing chores, queer couples manage to turn the home into a political space, quietly unsettling the heteronormative ideas of what gender and traditional family are supposed to look like (Kentlyn, 2008). As a liminal space, the queer household operates both as a private space for the quiet exchange of care and as a politically active site where the routines of domestic life challenge conventional ideas of gender labour (Gorman-Murray, 2008). Queering the home involves resistance to normative life trajectories through embracing what is perceived as a mess. This practice challenges hegemonic heteronormalization and creates alternatives to orderly existence (Manalansan and Martin, 2015). For instance, queer individuals - assigned female at birth (AFAB), in India, build and navigate intimate relationships within a society with heterosexual norms. This required subtle resistance and emotional labour to create safe, intimate, and political private spaces (Chakravarty, 2021).

Legal challenges continue to exist for queer individuals in India despite the decriminalisation in 2018. Legal protections were insufficient for addressing the specific privacy and safety concerns of queer women, particularly regarding live-in relationships and pressure toward heterosexual marriage, even after the ruling (Shukla, 2020). Despite transformative legal progress, a persistent discrepancy remains between India’s legislative changes and broader societal acceptance of queer individuals (Pandey and Kumar, 2023). This ongoing gap means that while legal rights may exist, the continued experience of social stigma and discrimination exacerbates mental health issues and contributes to social isolation within the community (Kottai, 2021). Queer movements must continually navigate the friction between globally dominant sexual expressions and the specific demands of local, situated identities (Bakshi and Dasgupta, 2019; Tellis, 2012). Even as activism and judicial observations have fundamentally reshaped the queer rights landscape, a divergence persists between legal reform and substantive social change (Shukla, 2022). Legislative backing is required to address the broader societal issues that continue to perpetuate mental health challenges and social isolation due to discrimination and stigma (Kottai, 2021).

Within this layered social and spatial politics, literary texts can be understood as cultural archives through which histories of queer intimacy become analytically accessible. Rather than simply reflecting society, literary works register historically specific social relations and what Williams (1977) terms “structures of feeling,” making them valuable sites for sociological inquiry. As Jameson (1981) argues, literary narratives function as socially symbolic acts that encode historical contradictions and lived experience, while Hall (1997) emphasises that representation actively produces cultural meaning rather than merely mirroring reality. Building on the historical recovery of same-sex desire undertaken by Vanita and Kidwai (2000) and Vanita’s (2024) novel *A Slight Angle* extends this archive through historical fiction, reconstructing how colonial institutions, domestic life, education, and everyday social interactions shaped queer intimacy. Rather than documenting historical events, the novel imaginatively represents the spatial, affective, and institutional conditions through which queer belonging was negotiated under colonial rule. As such, it provides a valuable literary site for examining how intimacy emerges through disciplinary, familial, and social structures.

While existing scholarship has documented the historical presence of queer lives in South Asia (Vanita and Kidwai, 2000), the spatial politics of queer belonging (Gopinath, 2005; Hazarika, 2022), and the ethical negotiations of intimacy in India (Dave, 2012), comparatively less attention has been given to how contemporary historical fiction can itself function as a cultural archive through which these negotiations become analytically visible. Rather than treating literary texts as fictional representations detached from social reality, this study adopts a literary sociological approach that understands fiction as a cultural form through which historical social relations become visible (Williams, 1977; Jameson, 1981). In doing so, the analysis contributes to literary sociology by demonstrating how historical fiction can function as a cultural archive through which institutional regulation, spatial organisation, and affective practices become sociologically visible. The study uses literary representation to examine social processes that continue to shape queer belonging across historical and contemporary India.

Collectively, this body of scholarship establishes that queer life in India cannot be understood through Euro-American theoretical models alone. Colonial governance, caste, religion, language, domesticity, and regional histories constitute interconnected social formations through which sexuality is organised. The present study extends this scholarship by examining how historical fiction functions as a cultural archive through which these institutional processes become sociologically visible.

## 3 Methods and materials

This study adopts an interpretive qualitative methodology situated within literary sociology and cultural studies, where literary texts are understood as cultural archives that refract historically situated social relations. The study employs textual analysis to examine how literary representation renders colonial institutions, social norms, and affective practices analytically visible (McKee, 2003). This approach follows traditions in literary sociology that understand narratives as sites where social structures, cultural meanings, and lived experiences are negotiated (Williams, 1977; Moretti, 2013). The objective of the study is not to verify the historical events represented in the novel but to examine how literary representation illuminates the organisation of colonial social life and its implications for contemporary understandings of queer belonging. The novel is therefore analysed as a literary representation of historically situated social relations rather than as documentary or empirical evidence.

The analysis proceeds through close textual reading of Ruth Vanita’s *A Slight Angle*, focusing on literary details such as minor gestures, queer glances, silences, and repetitions as sites of affective production (Sedgwick, 2003). This reading foregrounds the novel’s central paradox: disciplinary regulation simultaneously constrains and produces queer attachment. The analytical framework combines key concepts from queer theory to interpret the interplay of space, power, affect, and temporality within the novel. Foucault’s concepts of *disciplinary power* and the *dispositif* (apparatus) guide the interpretation of classrooms, domestic interiors, and other institutional spaces as overlapping sites where authority and intimacy converge (Foucault, 1978). These concepts are employed as analytical tools rather than universal explanations, and their application is grounded within South Asian scholarship demonstrating how sexuality in India is organised through colonial governance, caste hierarchies, religious difference, and regionally specific histories of intimacy (Vanita and Kidwai, 2000; Gopinath, 2005; Dave, 2012). Ahmed’s *queer phenomenology* extends this spatial analysis by providing a conceptual vocabulary for orientation and affect, demonstrating how bodily alignments, gestures, and movements choreograph queer desire and reroute attachments within these regulated spaces (Ahmed, 2006). Halberstam’s concept of *queer time* and Muñoz’s notion of *queer futurity* guide the temporal analysis of the novel by examining how non-heteronormative experiences challenge linear and normative temporal narratives (Halberstam, 2005; Muñoz, 2009). Halberstam’s concept elucidates how minor gestures produce queer intimacy, while Muñoz’s notion highlights how fleeting moments at liminal thresholds generate anticipation and endurance under conditions of constraint.

Taken together, these concepts are interpreted through South Asian queer scholarship that situates sexuality within colonial histories, caste, religion, regional spatialities, and everyday ethical negotiations (Vanita and Kidwai, 2000; Gopinath, 2005; Dave, 2012; Hazarika, 2022). This regional scholarship provides the interpretive context through which Foucauldian, phenomenological, and temporal concepts are applied throughout the analysis. Passages were selected through qualitative textual analysis guided by the study’s analytical framework (McKee, 2003). Selection prioritised scenes in which educational institutions, domestic environments, colonial infrastructures, bodily orientations, or temporal disruptions mediated interpersonal relationships. These passages were examined to explore how literary representations of space, institutional regulation, affective practice, and temporality illuminate broader sociological processes shaping queer belonging.

## 4 Discussion

Situated within India’s postcolonial socio-cultural landscape, *A Slight Angle* provides a literary representation through which broader social relations surrounding sexuality, institutional regulation, and belonging become analytically visible. The novel organises classrooms, homes, theatrical spaces, and transitional environments as sites where colonial power intersects with everyday practices of intimacy. Examining these representations allows the analysis to investigate how literary narrative reflects historically situated forms of social organisation that continue to influence contemporary queer belonging in India.

### 4.1 Pedagogical and private spaces of queer intimacy

Educational institutions function as spatial configurations where authority, intimacy, and desire converge. Space is socially produced through everyday practices and orientations (Lefebvre, 1991), while queer spatiality foregrounds how these orientations can be rerouted toward emotional attachments that exceed heteronormative regulation (Ahmed, 2006; Oswin, 2008). The relationship between Sharad and Abhik illustrates how pedagogical authority organises intimacy within colonial educational institutions. In the classroom, he internalises Abhik’s authority through constant self-monitoring, like adjusting his pronunciation, practising the gestures, and imagining Abhik’s gaze. The novel represents this institutional intimacy through Sharad’s everyday acts of self-monitoring. When his friend Muri asks him whom he will think of before sleeping, Sharad wonders how he could ever “tell him that it is Abhik’s face and body he sees in the mingling of dark and light.” He drops the “Sir” in his mind and even imagines that marrying Abhik’s sister could bind him closer to him, though not in the way he wants. He foreshadows the grammar corrections Abhik might make in his mind’s voice, even when he is alone, indicating a disciplinary intimacy in the absence of authority (Foucault, 1978; Kinkaid, 2019). The significance of this scene lies not only in Sharad’s emotional attachment but also in the way institutional authority becomes internalised as a mode of everyday self-regulation. The classroom reflects how educational institutions organise affective life alongside formal discipline. Correction becomes an eroticising medium because learning is already a libidinal relationship with authority (Britzman, 1995). Ahmed (2006) argues that social norms operate as straightening devices which, through the repetition of actions like “grammar correction,” shape our bodily orientations and desires, resulting in a complex queer attachment where disciplinary power is internalised. Sharad’s intimate desire is structured by pedagogy’s spatial and affective dimensions, showing how repetition of minor gestures generates what can be constituted as ‘queer spaces.’ Memory and embodied traces such as ‘the bruise on Sharad’s arm’ further anchor pedagogical authority within desire, symbolising how disciplinary intensities persist as affective residue (Freeman, 2010). The classroom operates as a social institution where disciplinary authority shapes interpersonal attachment through everyday practices of teaching, correction, observation, and self-regulation. As represented in the novel, these institutional processes reveal how colonial education simultaneously disciplined subjects and created the conditions through which queer relationality emerged.

The theatre rehearsal extends these pedagogical relations beyond the classroom, demonstrating how colonial educational practices continue to shape intimacy across adjacent institutional spaces. When Abhik asks Sharad to play Celia in *As You Like It*, casting becomes a spatial inscription of authority and recognition, more than a mere theatrical collaboration. Sharad is positioned within ‘Shakespeare’s cultural capital’ as a symbol of sophistication and colonial inheritance because of Abhik’s personal choice. This practice highlights how acquaintance with the plays secured elite status for Indians in the eyes of colonial masters (Trivedi and Bartholomeusz, 2005). As Ania Loomba and Martin Orkin argue, Shakespeare’s texts became part of a larger colonial project to assert cultural hegemony (Loomba and Orkin, 1998). This notion of a universal Shakespeare ‘loved’ by all Indians is clearly a colonial legacy, one that continues to influence postcolonial academic discourse (Singh, 1989). Intimate relations and sexual practices were central to the production and governance of colonial life (Stoler, 2006). Gender, particularly through the Shakespearean colonial tool, which is sustained through the repetition of seemingly natural performative acts (Butler, 1990), becomes unstable as Sharad embodies Celia. This theatrical discipline induces a ‘queer reorientation,’ which is prone to redirection, as Sharad’s gaze shifts to Abhik rather than the actor portraying Rosalind. This illustrates how a power dispositif (Foucault, 1978) can be subverted by queer desire (Vanita and Kidwai, 2000), while regulating posture and gesture. Even Sheela perceives this charged gay proximity before realising her own lesbian orientation later in her life and in the plot. The theatre operates as an institutional extension of the classroom, demonstrating how colonial cultural education organised bodies, performance, and affective relations within shared social space.

These institutional dynamics also extend into peer and family relationships, indicating that pedagogical discipline exceeds the formal boundaries of the classroom. Bechan’s warnings about the dangers of playing female roles and the family’s shame over the costume reiterate the stigma surrounding feminised embodiment. However, queer spatiality emerges precisely within and against these forms of regulation, where attachments survive in the interstices of disciplinary governance (Oswin, 2008). Spatial arrangements such as seating, rehearsals, and walking contribute to the possibility of intimacy, where “pauses between sentences, shared snacks, or casual interludes” become sites of affective intensity. These minor strategies, tactics, and negotiations (Halberstam, 2005) generate a libidinal relationship with authority (Britzman, 1995), where spatial control not just restricts but also creates the conditions for queer desire to emerge. Memory and embodied traces persist as affective residue (Freeman, 2010; Muñoz, 2009). These interactions indicate that family and peer networks participate in the regulation of queer intimacy alongside formal institutions, extending disciplinary authority beyond the classroom into everyday social life. In these ways, educational institutions become eroticized spaces where spatial control shapes the orientations through which queer desire emerges. Educational institutions therefore function as social environments where regulation and attachment are produced simultaneously. These interactions illustrate how institutional structures generate forms of queer belonging through ordinary pedagogical practices instead of functioning only as mechanisms of control.

Domestic space functions as a continuation of institutional surveillance, where family relationships regulate intimacy through everyday observation. When Abhik’s letter arrives via his sister, Kanta, Sharad locks himself away after noticing her searching look, capturing how the domestic interior acts as a space of internalised regulation where the gaze is pervasive (Lefebvre, 1991). The room functions as a ‘heterotopic space’ where a desired encounter is replayed through memory and anticipation (Foucault, 1986). Later, in the seaside home, Mrs. D’Souza eyes Rita and Sheela with some suspicion after they fall asleep together on the divan. For queer individuals, home is a place of both safety and intense observation (Tongson, 2011). Navigating this space involves a kind of ‘queer privacy,’ which is a constant practice of deciding what parts of oneself to show and what to keep hidden (Ahmed, 2006). This negotiation of visibility reflects what Brown (2005) describes as the spatial structure of the “closet,” where everyday environments organise when and how queer identities become visible. Domestic space functions as a social institution in which privacy, surveillance, and intimacy are continuously negotiated through everyday practices of family life.

Within these conditions of surveillance, intimacy is organised through minor affective practices that remain socially legible while avoiding direct visibility. Sharad’s careful handling of the letter on top of his unwillingness to wash away Abhik’s traces of touch shows how desire survives through affective residue (Freeman, 2010; Stoler, 2006). In Sheela and Rita’s narrative, intimacy unfolds through sensory engagement and everyday gestures. The moment Sheela enters Rita’s room and is struck by “the scent of her skin,” the space transforms into an archive of affect. Objects like a lamp, a mirror, or jewel cases can act as touchstones of memory, preserving the personal histories of feeling that Freeman (2010) calls “erotohistoriographies.” In intimate moments of foot pressing or sharing a bed, normal actions can become a secret, subversive language (Kentlyn, 2008). Within the home, these gestures of care are also coded ways of negotiating visibility and secrecy (Cvetkovich, 2003).

Educational and domestic spaces operate as social institutions that organise authority, intimacy, and affect simultaneously. Even as the power dispositifs, like those of the school, extend their corrective gaze into the home (Foucault, 1978), this constant pressure can produce moments of affective residue and intense connection that preserve queer histories (Freeman, 2010). Queer spatiality does not rely on finding pre-existing safe havens, but rather, it involves a continuous process of improvisational negotiation of boundaries (Gieseking, 2020) as individuals ‘re-route’ conventional orientations toward non-heteronormative attachments (Ahmed, 2006; Oswin, 2008). The narrative emphasises how these minor strategies, tactics, and negotiations (Halberstam, 2005) transform the seemingly ‘ordinary in form’ into something ‘subversive in function’ (Kentlyn, 2008). The creation of queer space is an ongoing process of affective world-making that reveals how institutions shape emotional life through everyday spatial practices (Cvetkovich, 2003). These interactions show that queer belonging emerges through the continuous negotiation of educational and domestic structures that configure social relationships.

Beyond the narrative itself, these representations illustrate how educational institutions function as social environments in which discipline and intimacy become mutually constitutive. The analysis therefore identifies schooling not only as a literary setting but also as a historically organised institution through which colonial authority shaped emotional life, social interaction, and queer belonging.

### 4.2 Colonial sites of queer desire

Colonial institutions organise language, education, caste, and cultural prestige as interconnected systems of social regulation. *A Slight Angle* represents these institutional processes through everyday interactions, making it possible to examine how colonial governance structured queer intimacy beyond formal legal regulation. Sharad reflects that “if English did not come to colonise andspread English, Sharad and Abhik would not have been able to communicate as they would be speaking Hindi and Bangla, respectively.” The English language was strategically imposed to create a class of persons Indian in blood and colour, but English in tastes, in opinions, in morals and in intellect (Macaulay, 1835, as cited in Spivak, 2008); simultaneously, this shared colonial language paradoxically enables cross-regional connections among the elite who use it as a common lingua franca (Pakir, 1991). Sharad’s desire for flawless English is not simply institutional compliance but a longing for recognition from Abhik, mediated through the colonial legacy itself. What is disciplinary becomes erotic, and what is alien becomes intimate! In Foucault’s words, “discourse transmits and produces power; it undermines and exposes it, renders it fragile and makes it possible to thwart it” (Foucault, 1978).

Language functions simultaneously as a mechanism of colonial governance and as a medium through which new forms of interpersonal attachment become possible. English similarly penetrates Sheela’s lesbian intimacy with Rita through aesthetics and consumer culture. Rita embodies colonial cosmopolitanism as a Bene Israel actress, her identity marked by gowns, perfumes, shoes, and cinematic glamour. Sheela, who raised herself in a nationalist environment of khadi and restraint, initially resists such markers, returning clothes “that were not khadi.” Yet their intimacy unfolds through precisely the gestures of applying lipstick, wearing rubber shoes, and having the Shalimar perfume lingering in the room. Colonial consumer modernity disciplines women’s bodies into spectacle, but in this process, desire finds its channel. These exchanges exemplify the aesthetic economy, where commodities derive value not simply from utility but through cultural valorisation, fostering social connections and intimacy through aesthetic forms (Entwistle, 2009). The act of exchanging perfume or clothing, while seemingly a material exchange within the aesthetic economy of colonial modernity, is fundamentally affective, illustrating how desire is shaped by movement and proximity as bodies and objects come into relation through ‘shared surfaces of feeling’ (Ahmed, 2006). Consumer culture therefore operates as a social process in addition to its aesthetic expression through which colonial modernity organises intimacy, identity, and belonging.

Caste and religion further structure queer belonging where Sharad frames his longing for Abhik in matrimonial terms, noting that Abhik’s caste identity as Kayastha, “although a Roy,” aligns with his own Mathur identity, “as if their marriage is getting arranged.” Dirks (2001) argues that the modern understanding of caste is not an ancient survival but was fundamentally a product of British colonial knowledge and administration, used to categorise and control Indian populations. Even in desire, Sharad measures intimacy against caste equivalence, demonstrating how queer longing is imagined within the frameworks of colonial scripts of endogamy. Kanta’s relationship with Robin, a Christian Singh, parallels this dynamic as both attachments confront social and religious prohibitions, with Sharad’s queerness disciplined by pedagogy and caste, while Kanta’s heterosexuality is constrained by religion. Historically, sexual norms in India have been policed through caste and religious endogamy to maintain caste purity (Chakravarti, 1993), and Brahmanical patriarchy continues to subjugate transgender and gender non-conforming individuals whose identities challenge these systems of reproduction and hierarchy (Ponniah and Tamalapakula, 2020; Upadhyay and Bakshi, 2020). These relationships mark how caste and religion regulate queer belonging through the same institutional logics that organise heterosexual respectability.

Rita, as a Jewish woman, has intimacy with Sheela, which unsettles nationalist ideals of purity, illustrating that lesbian belonging in colonial Bombay is shaped by religious difference as well. Their shared household, with Mrs. D’Souza present as caretaker, becomes a microcosm of cosmopolitan affiliation, which Sheela describes as “a little side-pool screened by rushes, while beyond, the current flowed on as usual.” Belonging here is reimagined outside sanctioned forms of marriage, emerging through secrecy, care, and domesticity. This exemplifies “transculturation,” a space of mutual transformation in which cultural difference becomes the ground of affiliation (Ashcroft, 2001). While Sheela’s intimacy evolves through domestic aesthetics, Sharad’s desire finds expression in the intellectual and performative world of English literature, particularly through Shakespeare.

Shakespeare operates as the central conduit for Sharad and Abhik’s bond, transforming the canonical texts of colonial education into a space for queer intimacy. The introduction of English literature, particularly canonical authors like Shakespeare, served as an effective form of ‘political control’ and ‘cultural assimilation’ in British India (Viswanathan, 1989). During their (Abhik and Sharad) long walks, they talk about Shakespeare’s plays *As You Like It*, *Hamlet*, *Lear*, and *The Merchant of Venice*, exploring characters and plots. These conversations become a site for intimacy and desire, where they can bond over their love for literature beyond the restrictions of the classroom. The classroom and the stage become intimate terrains, reconfigured by Sharad and Abhik through performance and conversation, which creates the alternative space that is queer, within heteronormalised space. Shakespeare functioned as a disciplinary tool in colonial classrooms, symbolising refinement and authority, a dynamic whereby he became a symbol of English cultural superiority (Loomba and Orkin, 1998). Shakespeare’s influence goes beyond their conversations into the theatre, where Abhik assigns roles for the play. Following the colonial tradition, he has the boys perform the female characters, including Sharad as Celia. This practice continues the colonial style of theatre in Indian schools. The colonial script itself becomes a site of aesthetic transformation where meaning is renegotiated, as postcolonial resistance is most effective when it is transformative, when it takes the language of power and makes it work in the service of the powerless (Ashcroft, 2001). Shakespeare functions as a literary reference within the novel but as an institutional technology through which colonial education organised cultural legitimacy. This illustrates that literary canon formation also shaped interpersonal relations, emotional attachment, and social hierarchy.

Sheela’s queer intimacy with Rita is similarly inflected by another colonial apparatus, ‘cinema’ (Chowdhry, 2000). Rita’s “swaggering” in a waistcoat and tie, her theatricality in daily gestures, blur the line between performance and desire. Sheela remarks that watching Rita was “as good as a moving picture.” The colonial institution of cinema disciplines female bodies into spectacle operating within what Laura Mulvey terms ‘erotic spectacle,’ where the woman holds the look, plays to and signifies male desire (Mulvey, 1975), but this visual regime also produces space for lesbian intimacy in the act of watching. As Foucault (1977) would put it, the cinematic gaze exemplifies how mechanisms of surveillance and power/knowledge relations actively produce the very subjects they aim to regulate, giving rise to new forms of identity and desire, making the individual and the knowledge belong to that production. Like Sharad in Shakespeare’s play, Sheela moves within a world shaped by performance, where the aesthetics of colonial modernity create the conditions for queer desire to emerge. The instability of aesthetic value clarifies this dynamic: cinema, like fashion, is an aesthetic economy where recognition depends on constantly shifting signs of prestige and desirability (Entwistle, 2009); this spectacle, structured by colonial modernity, functions simultaneously to discipline bodies and generate queer desire (Chakravarti, 1993; Puar, 2007). The instruments of power, English education, Shakespearean literature, cinema, and caste systems, become the very mediums through which desire is articulated and sustained.

These literary representations illuminate broader colonial processes through which language, education, caste, and cultural prestige organised access to social legitimacy. The novel therefore functions as a sociological representation of colonial institutions whose influence continues to shape unequal distributions of queer visibility and belonging in contemporary India.

### 4.3 Queer temporal and liminal spaces

Time and transitional space function as social conditions through which recognition, anticipation, and belonging are organised. *A Slight Angle* represents these conditions through recurring moments of waiting, movement, memory, and delayed communication, providing a basis for examining how queer temporal experience develops within institutional constraint. Sharad’s reflections on his experiences with Abhik exemplify the elasticity of time, or more accurately, ‘queer temporality’. He recalls, “…On the train, we stayed up late, playing cards. When I awoke at dawn, the rest were still asleep. I sat at the window, watching the fields glide toward me like the present rapidly become the past.” Time is experienced not as linear but as a fluid through past, present, and anticipated future converge in Sharad’s perception. The mundane act of watching fields sliding by becomes charged with memory, longing, and a heightened awareness of relational presence. As Halberstam (2005) puts it, queer uses of time and space develop, at least in part, in opposition to the institutions of family, heterosexuality, and reproduction. Sharad experiences time in a new way, where desire makes everyday moments feel deep and significant. Temporal experience is therefore constituted as a socially organised dimension of queer life rather than merely an individual emotional response.

The bodily dimension of queer temporality is powerfully evident in Sharad’s attentiveness to sensation and proximity, particularly his olfactory engagement with Abhik, where he wanted to lean closer and breathe in “the smell of mustard oil and something else that was undefinable” until he realised it was not unlike the smell of his own unwashed body. This sensory moment resists the forward march of normative time, creating a suspended, intensified present. The blending of scents and the blurring of self/other boundaries exemplify the ‘deviant chronopolitics’ where immediate bodily pleasure actively challenges and reorders standard social timelines (Freeman, 2010). This sensory attunement acts as an affective orientation within shared space, aligning with Sara Ahmed’s observation that orientations shape not only how we inhabit space, but how we apprehend this world of shared inhabitance, as well as ‘who’ or ‘what’ we direct our energy and attention toward (Ahmed, 2006). The queer temporal experience is amplified by the underlying regulatory conditions, producing subjective, suspended, and affectively charged moments of intimacy within a world shaped by social constraints (Foucault, 1978).

Names, gestures, and minor affective acts also operate as temporal markers within Sharad and Abik’s relational world. Sharad savours the way Abhik’s lips caressed his name, “rounding the A as Bengalis do, making it his own.” The careful attention to the pronunciation of a name transforms a quotidian act into a temporally saturated moment of intimacy. Time here is neither measured in hours nor days. Still, in relational intensity, the lingering articulation of a name becomes a unit of queer time, wherein desire, recognition, and affect coexist. The intense focus on such minor acts and linguistic nuances is central to queer experience, where details function as significant markers of connection and understanding (Sedgwick, 1990). These are the minor strategies, tactics, and negotiations that shape life narratives different from those prescribed by the dominant culture (Halberstam, 2005). Such emotions operate as sticky forces that produce social worlds and attach feeling to objects and bodies (Ahmed, 2004). This emphasis on navigating compromised landscapes through affective value is a key concept for understanding how individuals remain attached to fantasies of the good life, even amid deteriorating social conditions (Berlant, 2011). Similarly, Sharad’s reflection on jewellery, “…I thought of jewellery as something that goldsmiths make, but I was wrong, clearly. Worlds are breaking down. Perhaps they were always half-broken in cities,” conveys temporal disjunction. This unstable aesthetic economy speaks to material objects that resist normative functional timelines, instead embodying a suspended, affective history, a site of erotohistoriography (Freeman, 2010). Ordinary structures of labour, craft, and urban life are destabilised in Sharad’s perception, and in this instability, time itself feels suspended and reoriented around affective experience.

Queer temporality also emerges through deferred communication, absence, and anticipation. When Sharad meets Abhik after so long, he just greets him as if nothing had happened, but he was frozen inside. The encounter is brief, but the internal experience of time expands, dominated by anticipation, memory, and desire. Similarly, when Sharad reads Abhik’s letters and runs toward him, tears streaming down his cheeks, the delay in their communication intensifies the emotional and temporal experience. The letters mediate time and space, creating moments of longing, recognition, and affect that overlay chronological time. This focus on yearning and potentiality, rather than the ‘here and now’ of immediate gratification, aligns with ‘queer futurity’, which posits that queerness is a longing that propels us onwards, beyond romances of the negative toward a utopian horizon of possibility (Muñoz, 2009). This frozen moment, dominated by internal emotional damage, reflects the backward feelings that serve as an index to the ruined state of the social world (Love, 2009). The affective connection across absence functions as a form of touch across time, allowing contact between subjects separated by temporal and social distance (Dinshaw, 1999). The tears and freezing are manifestations of a queer orientation toward both a painful past and a future possibility that is not yet here (Love, 2009; Muñoz, 2009). These deferred encounters illustrate how institutional constraints extend beyond physical space into temporal experience. Delay itself becomes a socially organised condition through which queer relationships are sustained, interrupted, and remembered.

Alongside queer temporality, the narrative foregrounds liminal spaces and encounters, emphasising in-between sites where affect and intimacy unfold. Sharad recounts entering a small gate that opened into dense thickets, where a path so narrow forced him and Abhik to brush against each other as they walked. The ridge becomes a liminal site, that is physically transitional, socially ambiguous, and affectively charged. Affect circulates through these spaces, where physical closeness on a narrow path mediates both desire and relational intensity (Ahmed, 2004). Liminality is further visible in domestic and transactional spaces, such as during “bargaining in Bangla” for “saris,” when Sharad “sat across the room” to watch Abhik “stretch his limbs.” The act of observing gestures in a semi-public space situates desire within thresholds between visibility and concealment, which can be viewed through the concept of liminality as a zone of ambiguity where normative social structures are temporarily suspended (Turner, 1969). Bhabha (1994) further theorises this ‘in-between’ state as a crucial ‘third space’ of hybridity, where colonial power dynamics can be subverted. The private reclamation of space also operates as a form of liminality. Sheela “exulted in having the house to herself” and moved about “without haste” just “as she was beginning to miss Rita,” illustrating how temporal and spatial suspension creates conditions for reflection and longing, and how queer temporality and liminality become mutually constitutive in shaping affective life.

Liminality also intersects with memory and trauma, revealing how past experiences shape psychological thresholds of consciousness. Sheila’s reflection on her father, “touching her very long back, like when she was a child,” which Sheila does not even remember, exemplifies how memory constitutes an ‘in-between’ temporal and psychological space. Trauma, in this sense, is an event ‘not fully known in the first instance,’ which creates a breach in the mind’s experience of time, leading to a complex relationship with the past (Caruth, 1996). The concept of liminality can be applied to matters of memory in various productive ways, helping to conceptualise the dynamic and often blurry lines between the remembered and the forgotten (Mueller-Greene, 2022). The presence of ‘negative backward feelings’, such as the shame or damage surrounding trauma, often serves as an index to the ruined state of the social world, a crucial aspect of marginalised experience (Love, 2009). Similarly, Sheela’s rare guilt for Abhik, considering if a “change would be good for him,” highlights liminality as both spatial and emotional. The intimate sensory space, when Sheela describes Rita’s body smell, “overlaid with others. Jasmine, rose, sandalwood, much more,” emphasises the fleeting nature of liminal encounters, where affect and attention are elevated, yet socially constrained. These in-between moments, whether alleyways, intimate rooms, or sensory perceptions, are central to the narrative’s articulation of queer experience. In its depiction of fleeting encounters and sensory awareness, intimacy is situated within the fluidity of time and the permeability of space. These subtle negotiations transform constraint into continuity, illustrating that queer belonging endures through affective resilience rather than overt liberation. These temporal and spatial representations illustrate social conditions in which queer lives develop through uncertainty, delay, and negotiated recognition. The analysis highlights how literary depictions of time and liminality illuminate historically organised conditions that continue to shape queer belonging beyond the colonial period.

## 5 Conclusion

Reading *A Slight Angle* as a cultural archive through a literary sociological lens demonstrates how colonial institutions organised everyday practices of intimacy, mobility, and belonging in ways that continue to resonate within contemporary queer life in India. Across domestic, educational, and liminal spaces, the novel reveals how everyday negotiations of visibility, secrecy, and affect shape queer belonging under conditions of regulation. The secrecy surrounding Sharad’s letters, the careful management of visibility within domestic spaces, and the reliance on small gestures of intimacy mirror the strategies through which many queer individuals continue to navigate family expectations and social surveillance. Although legal frameworks have shifted with the decriminalisation of same-sex relations, the absence of marriage rights and the persistence of normative family structures mean that queer intimacy often remains confined to negotiated spaces of privacy. The novel therefore traces a historical genealogy of these negotiations, demonstrating that contemporary queer spatial practices remain embedded in longer histories of colonial regulation, moral discipline, and social hierarchy. These negotiations persist not as direct historical continuities but as emotional and spatial legacies that continue to inform contemporary queer belonging within India’s uneven legal recognition and enduring social hierarchies.

The analysis demonstrates that queer life is sustained through the reconfiguration of time, space, and affect, drawing on Halberstam’s notion of queer time, Ahmed’s concept of orientation, and Foucault’s analysis of disciplinary power. The temporal disruptions and suspended moments across Sharad and Abhik’s encounters, including delayed letters, lingering glances, and vivid memories, illustrate how queer desire resists linear, heteronormative progressions of intimacy. Likewise, the domestic and pedagogical interiors that shape Sheela and Rita’s relationship demonstrate how private intimacy transforms regulated spaces into arenas of affective negotiation. These layered gestures constitute a queer archive of everyday acts that challenge colonial and social order while sustaining intimacy within conditions of constraint. The analysis further suggests that power and desire are mutually constitutive within the novel: the very mechanisms of colonial discipline, education, and domestic regulation that constrain intimacy also produce the conditions through which queer life endures. Educational institutions, domestic interiors, and liminal spaces collectively demonstrate how belonging is organised through ordinary social practices rather than exceptional moments of resistance.

Reading the novel as an affective archive of colonial intimacy reframes queer historiography in South Asia, not as a narrative of rupture and liberation but as one of endurance, memory, and affective survival. The seaside bungalow, hostel room, and letter function as thresholds between visibility and secrecy, illustrating how intimacy emerges through the careful negotiation of constraint as much as through moments of freedom. Rather than presenting fiction as documentary evidence, this study demonstrates the value of literary sociology for examining historically organised forms of social life. By treating literary representation as a cultural archive of institutional relations, the paper shows how historical fiction can contribute to sociological understandings of queer belonging, colonial modernity, and the spatial organisation of intimacy in contemporary India.

## Data Availability

The original contributions presented in the study are included in the article/supplementary material, further inquiries can be directed to the corresponding author.
